# Association between brown adipose tissue activity and clinical outcomes in melanoma: a retrospective PET/CT analysis

**DOI:** 10.1016/j.iotech.2026.101600

**Published:** 2026-07-23

**Authors:** P. Toivanen, P. Ketola, T. Vahlberg, K.A. Virtanen, M. Sundvall, J. Raiko

**Affiliations:** 1 Department of Oncology and FICAN West Cancer Center, University of Turku and Turku University Hospital, Turku, Finland; 2 MediCity Research Laboratory, Turku PET Centre, University of Turku, Turku, Finland; 3 Department of Biostatistics, University of Turku, Turku University Hospital, Turku, Finland; 4 Turku PET Centre, Turku University Hospital, Turku, Finland; 5 Cancer Research Unit, Institute of Biomedicine, University of Turku, Turku, Finland; 6 Department of Clinical Physiology and Nuclear Medicine, Kanta-Häme Central Hospital, Hämeenlinna, Finland

**Keywords:** brown adipose tissue, melanoma, positron emission tomography

## Abstract

**Background:**

Brown adipose tissue (BAT) activity has been suggested to play a role in cancer progression. Previous studies have shown that BAT activity is higher in patients with cancer, and that BAT volume is a predictor of tumour recurrence and mortality in patients with cancer, but the data on melanoma are limited.

**Patients and methods:**

Here, we re-analysed 2-fluoro-2-deoxy-D-glucose positron emission tomography-computed tomography (FDG–PET–CT) images from 135 patients with cutaneous melanoma treated at Turku University Hospital between 2012 and 2021 to assess associations among BAT, melanoma progression, patient survival, and patient weight. We applied a three-stage universal BAT threshold definition using predetermined standardised uptake value thresholds of 0.8, 1.0, and 1.2 g/ml, given the retrospective nature of our study. Of the 135 patients (81 men and 54 women; median age 61 years, interquartile range 54-71), 40 (29.6%), 24 (17.8%), and 19 (14.1%) were BAT-positive at the 0.8, 1.0, and 1.2 g/ml thresholds, respectively.

**Results:**

Our results showed that patients with active melanoma on FDG–PET–CT imaging were more frequently BAT-positive at the 0.8 threshold (*P* = 0.026) and 1.0 threshold (*P* = 0.016). We also found a significantly higher BAT volume among patients who survived the observation period (0.8, 1.0, and 1.2 g/ml thresholds; *P* = 0.018, *P* = 0.038, and *P* = 0.571, respectively) and those who did not relapse (0.8, 1.0, and 1.2 g/ml thresholds; *P* = 0.631, *P* = 0.012, and *P* = 0.030, respectively).

**Conclusions:**

No association between BAT positivity and relapse-free survival or overall survival was observed at any threshold. Although higher BAT volumes were observed in subgroups of patients who survived or did not relapse, these findings were not supported by survival analyses and should be considered exploratory.

## Highlights

• Previous studies have shown that BAT activity is higher in patients with cancer, and that BAT volume is a predictor of tumour recurrence and mortality in patients with cancer.
• No association between BAT positivity and relapse-free melanoma survival or overall survival was observed.
• Higher BAT volumes were observed in subgroups of patients who survived or did not relapse.

## Introduction

Cutaneous melanoma is a malignant tumour that arises from pigment-producing cells of the skin, accounting for 90% of deaths associated with cutaneous tumours.^1^ Worldwide, ∼330 000 cases of cutaneous melanoma are diagnosed annually, resulting in ∼58 000 deaths each year.^2^ Cutaneous melanomas exhibit substantial heterogeneity and transcriptional plasticity,^3^ but approximately half harbour a clinically relevant activating mutation in the signalling protein BRAF, which is amenable to therapeutic targeting.^4^ Melanomas are considered highly immunogenic tumours due to their high mutational burden,^5^ allowing for the effective utilisation of immune checkpoint therapies. Primary melanomas are treated with surgery.^6^^,^^7^ Treatment beyond surgery depends on the disease stage, which is usually determined by histopathological assessment of the primary tumour and sentinel lymph node biopsy. During the observation period of this study, the treatment landscape of melanoma has shifted, with the advent of BRAF-targeted therapy and immunotherapy superseding chemotherapy. Currently, for high-risk cases, immune checkpoint inhibitors, BRAF-targeting therapy, or, in certain situations, radiotherapy are used as adjuvant therapy.^8^ For metastatic melanoma, immune checkpoint inhibitors are used, and in patients with BRAF mutations, BRAF pathway-targeted therapies are a treatment option, but only 30%-50% of patients survive >5 years.^8^

Brown adipose tissue (BAT) is heat-producing adipose tissue that achieves thermogenesis via uncoupling protein 1 (UCP-1)-mediated uncoupling of the electron transport chain; it is most prevalent in neonates, with its volume decreasing with age.^9^ Previous studies have shown that residual BAT activity in adulthood, most often in the supraclavicular or paraspinal regions, is associated with high insulin sensitivity^10^ and lower obesity parameters.^11^ Currently, [^18^F]2-fluoro-2-deoxy-D-glucose positron emission tomography-computed tomography (FDG–PET–CT) imaging is the most widely used method for quantifying human BAT functional activity.^12^ In addition to classical BAT, thermogenesis-capable adipocytes, also known as brown-in-white (brite) or beige adipocytes, exist. Beige adipocytes are located in white adipose tissue (WAT) deposits and are activated by various stimuli, such as cold exposure or sympathetic nervous activity. Like brown adipocytes, brite/beige adipocytes utilise UCP-1 for thermogenesis.^13^

Emerging evidence suggests that BAT plays a role in cancer. BAT activity has been shown to be significantly higher in patients with cancer.^14^ Likewise, a study comparing patients with BAT-positive cancer with age-, sex-, and body mass index (BMI)-matched individuals without BAT-positive cancer reported significantly greater BAT volumes in patients with cancer.^15^ Moreover, BAT volume, assessed using routine positron emission tomography-computed tomography (PET–CT), has been shown to be a predictor of tumour recurrence and mortality in patients with cancer, independent of other factors that may influence BAT activity.^16^ Potential cancer-promoting features of BAT may include the synthesis and secretion of angiogenic and growth factors associated with BAT activation, thereby promoting tumour angiogenesis, growth, and metastatic dissemination.^17^^,^^18^

Furthermore, BAT may influence cancer mortality through its proposed association with cancer-associated cachexia (CAC). CAC affects ∼60%-80% of patients with advanced disease and directly causes up to 20% of cancer deaths.^19^ Numerous mouse models have shown that increased thermogenic activity in adipose tissue, due to increased BAT activity and increased browning of adipocytes in WAT, contributes to elevated energy expenditure and weight loss during CAC.^20^, ^21^, ^22^ Loss of the transcriptional co-regulator PRDM16, which is essential for the browning process of adipocytes, ameliorates adipose wasting in tumour-bearing mice,^23^ and blocking β-adrenergic receptors, which are essential for thermogenesis in adipose tissue, has similar effects.^22^ Nevertheless, emerging clinical evidence has raised questions regarding the hypothesis that BAT activation plays a causal role in CAC. In their retrospective longitudinal analysis of 238 patients, Becker et al.^24^ evaluated subsequent PET–CT imaging studies and found no significant associations between changes in cancer burden, BAT activation, and BMI.

Different types of cancer are associated with varying prevalences of active BAT, although the reason behind the disparity remains unclear.^25^ Supporting this, a study by Cao et al.^26^ showed that the prevalence of BAT activity was threefold greater in patients with breast cancer than in age- and body weight-matched patients with other solid tumour malignancies, with a particularly marked difference in women aged ≤55 years.

Currently, limited evidence exists regarding the prevalence of BAT in melanoma patients and its potential association with melanoma progression and outcomes. Melanoma presents a unique opportunity to study BAT and its potential association with cancer, as PET–CT imaging is routinely used for staging in many clinics, enabling investigation of BAT without requiring additional imaging or radiation exposure. In this study, we re-analysed FDG–PET–CT images from 135 patients with cutaneous melanoma to investigate the effects of BAT activity on melanoma characteristics, disease progression, patient survival, and patient weight. We aimed to explore whether BAT positivity and greater BAT volume are associated with more aggressive disease status, as indicated by more advanced disease stage at diagnosis, melanoma activity on PET–CT, faster disease progression, higher melanoma-specific mortality, and lower patient weight.

## Materials and methods

### Patients

The cohort included patients diagnosed with cutaneous melanoma at Turku University Hospital between 2012 and 2021 who had undergone FDG–PET–CT imaging as part of their diagnostic workup. Patients with concurrent malignancies other than melanoma and conflicting medications were excluded. Auria data services were used to identify patients diagnosed with melanoma at Turku University Hospital who subsequently underwent PET–CT imaging. Relevant clinical data were manually extracted from the electronic patient records of Turku University Hospital. Patients for whom complete clinical data were unavailable were excluded. Additionally, cases of noncutaneous melanomas, patients with concurrent malignancies other than melanoma at the time of PET–CT imaging, and those whose melanoma diagnosis occurred before 2012 were excluded. The medications prescribed at the time of melanoma diagnosis were reviewed, and patients receiving β-blocker, benzodiazepine, and nonbenzodiazepine therapies were excluded due to the BAT-suppressing effects of these medications. Furthermore, patients receiving mirabegron therapy were excluded due to its potential BAT-activating effect.

### Measurement of BAT activity on PET–CT images

PET–CT images of the remaining patients were reviewed for the presence of BAT by two investigators under the supervision and guidance of an experienced clinical nuclear medicine physician. PET–CT studies were conducted in a clinical setting; thus, patients underwent imaging at ambient room temperature, and imaging conditions were not standardised. In particular, ambient room temperature was neither controlled for nor recorded, and no data were available on prior cold exposure or patient shivering. Image analysis was conducted using the Carimas image viewer software for Windows, version 2.10.0.0 (PET Centre, University of Turku, Turku, Finland).^27^ BAT was identified by increased 2-fluoro-2-deoxy-D-glucose (FDG) uptake on positron emission tomography (PET), measured in a region corresponding to adipose tissue on a computed tomography (CT) scan. Regions of interest (ROIs) were manually identified on the CT images of the supraclavicular, cervical, axillary, paravertebral, and perirenal adipose deposits on the left and right sides based on visual inspection of anatomical landmarks. Resultant ROIs were filtered to exclude voxels outside the radiodensity range of –190 to –10 Hounsfield units (HUs), corresponding to the expected radiodensity of adipose tissue. The standardised uptake value (SUV) was calculated as the ratio of a voxel’s radioactivity concentration to the decay- and body weight-corrected injected radiotracer dose. Subsequently, the minimum, maximum, and mean SUVs for each ROI were measured, along with the volume of each ROI. Each ROI containing voxels with SUVs exceeding the predetermined thresholds of 0.8, 1.0, and 1.2 g/ml underwent thresholding. Voxels with SUVs below the respective thresholds were excluded, and the maximum, minimum, and mean SUVs, as well as the total volume of the remaining voxels, were recorded for each threshold.

Paravertebral and perirenal measurements were excluded from the analysis due to confounding factors, including renal tracer accumulation, FDG uptake by the paraspinal musculature, and aortic activity, which interfered with accurate assessment of BAT activity. The large voxel size of the PET–CT images relative to the small size of the adipose deposits in these regions introduced significant measurement error, warranting their exclusion from the analysis.

In addition to BAT measurements, the metabolic activity of WAT was measured in subcutaneous abdominal fat as an exploratory surrogate to assess the potential magnitude of systemic influences on SUV measurements (e.g. inflammation, metabolic stress, and other confounding factors).

BAT activity was investigated across three categories based on predetermined SUV thresholds of 0.8, 1.0, and 1.2 g/ml. For each category, a patient was deemed BAT-positive if any of the six radiodensity-filtered ROIs contained voxels with SUVs exceeding the corresponding threshold. Consensus criteria recommend a lean body mass (LBM)-adjusted SUV cut point.^28^ However, this approach was not feasible in our study due to incomplete patient anthropometric data. Specifically, LBM-corrected SUV thresholds could not be applied because neither LBM nor the variables required for estimation were consistently available. Patient height was not systematically recorded at the time of PET–CT imaging, precluding the use of validated formulas (e.g. Janmahasatian equation) for LBM estimation. Therefore, we pragmatically applied fixed SUV thresholds (0.8, 1.0, and 1.2 g/ml), as these values span the range reported in prior studies^29^ and approximate the consensus criterion under typical LBM assumptions. The use of multiple thresholds allowed assessment of the robustness of the findings across SUV cut-offs and accounted for variability between imaging systems.^28^^,^^29^ BAT volume was calculated as the sum of the volumes of the thresholded ROIs at each threshold and was reported in mm^3^. Maximum SUV (SUV_max_) was defined for each patient as the highest recorded SUV across the six ROIs included in the analysis.

### Clinical data collection

Data were manually collected retrospectively from the electronic medical records of Turku University Hospital. The following demographic and medical history data were extracted: patient date of birth, patient sex, date of patient death, date of diagnosis, date of most recent oncology contact, date of most recent health care contact concerning melanoma, histopathological characteristics of the primary tumour, sentinel lymph node biopsy results, results of imaging studies, dates of metastases and recurrence diagnoses, patient medications at the time of diagnosis, dates of diagnoses of other malignant diseases, dates of PET–CT imaging, and clinical radiology reports for PET–CT imaging. The patients’ weights at the time of imaging were obtained from measurements taken during PET–CT radiotracer administration. Disease staging at diagnosis was recorded using the eighth edition of the American Joint Committee on Cancer (AJCC) melanoma staging system, based on the histopathological characteristics of the primary tumour, sentinel lymph node biopsy results, and imaging studies conducted at the time of diagnosis. BRAF mutation status was obtained from pathologists’ reports of primary tumour analysis for patients with available BRAF mutation information.

Melanoma activity status at the time of imaging was determined from the original clinical radiology report of the analysed PET–CT and classified as either no evidence of melanoma activity or evidence of melanoma activity, excluding equivocal reports from the analysis.

The observation period was defined as the time from the first oncologist visit to the date of the last recorded health care contact, the end of oncology contact, or patient death, whichever was applicable. Cases of local disease at the time of PET–CT imaging were selected from the study population based on the following criteria: disease stage at diagnosis < IV and no metastasis detected before or within 1 month after PET–CT imaging. For patients with local disease at the time of PET–CT imaging, progression-free survival was defined as the time from diagnosis to the detection of the first metastasis or to the end of the observation period if no metastasis was observed.

### Statistical analysis

All statistical analyses were conducted using SPSS Statistics for macOS, version 29.0.2.0 (IBM Corp., Armonk, NY). Age at diagnosis, observation period duration, time from diagnosis to metastasis detection, SUV_max_, BAT volume, and patient weight were treated as continuous variables. Patient sex, BRAF mutation status, and melanoma activity on PET–CT were coded and treated as categorical variables. Disease stage at diagnosis was initially collected in subgroups according to the AJCC Eighth Edition melanoma staging system but was subsequently grouped into main categories for analysis due to the sample size.

Data are shown as mean ± standard deviation (SD) for normally distributed data and as median (interquartile range [IQR]) for non-normally distributed data. *P* values < 0.05 were considered statistically significant. Multiplicity adjustment to *P* values was not carried out due to the exploratory nature of this research.

Continuous variables were tested for normality using the Shapiro–Wilk test. Associations between categorical variables were examined using the chi-square test or Fisher’s exact test, as appropriate. For the analysis of continuous variables between two groups, the independent samples *t*-test or the Mann–Whitney *U* test was used, depending on the data distribution. The Kruskal–Wallis test was used for comparisons of more than two groups. Spearman’s rank correlation was used to assess relationships between continuous variables. Kaplan–Meier analysis and log-rank tests were used to evaluate melanoma survival and progression-free survival between BAT-positive and BAT-negative patients. Cox proportional hazards regression was used to perform a multivariable analysis of overall survival and progression-free survival. The proportional hazards assumption was tested using time-dependent covariates.

### Ethical considerations

This study was supported by the ethical committee of Hospital District of South-West Finland and permitted by the Hospital District of South-West Finland (T11/016/21). All data were collected, stored, and handled in a manner that meets the General Data Protection Regulation set by the EU and the Secondary Use Act 552/2019. The data are not publicly available due to privacy restrictions and regulations mandated by the Secondary Use Act.

## Results

### Patient cohort and characteristics

**Figure 1:**
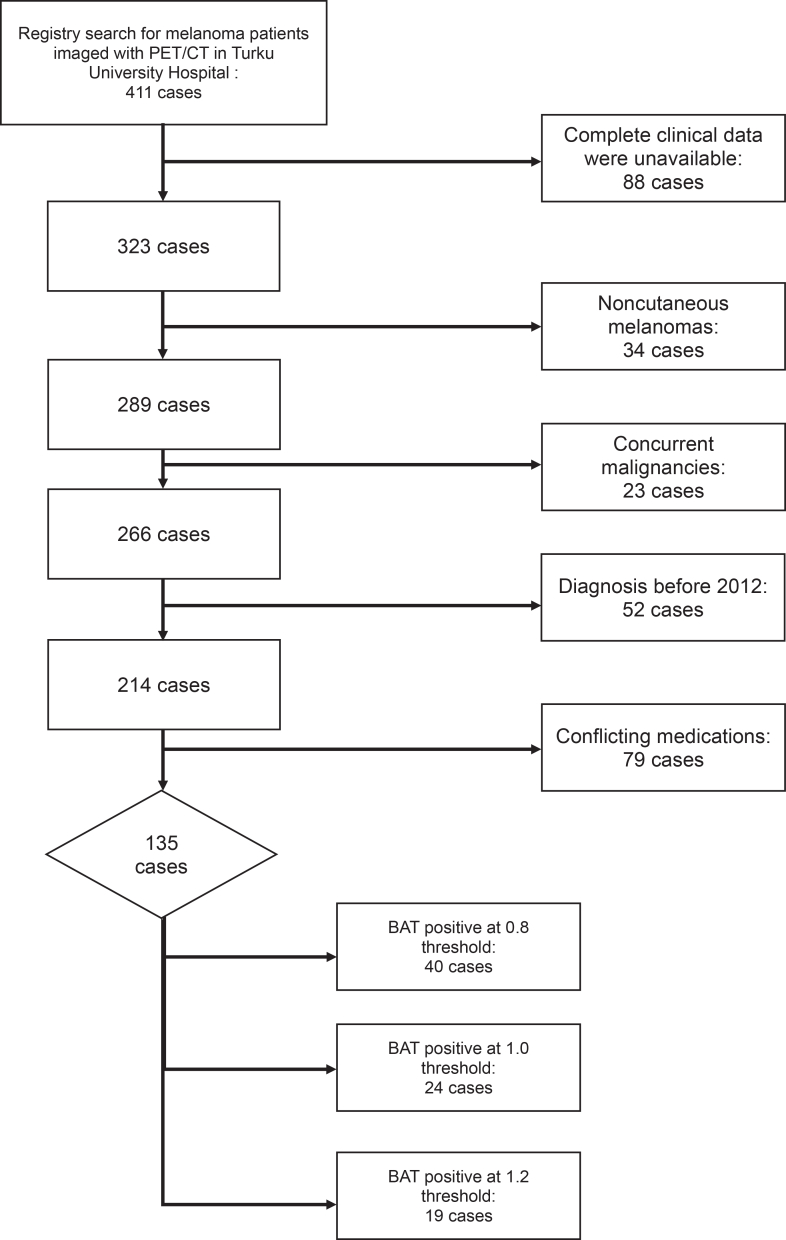
**Patient inclusion and exclusion flow diagram.** BAT, brown adipose tissue; PET–CT, positron emission tomography-computed tomography.

All patients diagnosed with melanoma at Turku University Hospital between 2012 and 2021 who underwent PET–CT imaging were included in our cohort (*N* = 411). Relevant clinical data were then manually extracted from electronic patient records. Patients for whom complete clinical data were unavailable (*n* = 88), who had noncutaneous melanomas (*n* = 34), had concurrent malignancies other than melanoma at the time of PET–CT imaging (*n* = 23), or who were diagnosed for the first time before 2012 (*n* = 52) were excluded. Patients receiving β-blocker (*n* = 59), benzodiazepine (*n* = 14), nonbenzodiazepine (*n* = 4), or mirabegron (*n* = 2) at the time of melanoma diagnosis were also excluded from the analysis due to the BAT-suppressing effects of these medications, resulting in a final population of 135 patients (Figure 1).

Of the 135 patients included in the analysis, 81 were men, and 54 were women. The median age at diagnosis was 63 years (IQR 54-71, range 19-83). The mean body weight for all patients at the time of PET–CT imaging was 83 kg (SD 16.5, range 46-134). At diagnosis, 120 patients had stage I, II, or III disease, while 15 patients had stage IV disease. BRAF mutation status information was available for 53 patients, of whom 34 were BRAF-positive and 19 were BRAF-negative. The median length of the observation period was 45 months (IQR 21-60, range 4-112). Patient characteristics for all patients and according to disease stage are summarised in Table 1. Adjuvant therapies received by patients with local disease at the time of PET–CT imaging are summarised in Supplementary Table S1, available at https://doi.org/10.1016/j.iotech.2026.101600. Treatments for patients with metastatic disease at the time of PET–CT imaging are summarised in Supplementary Table S2, available at https://doi.org/10.1016/j.iotech.2026.101600, and the number of patients by line of systemic therapy is summarised in Supplementary Table S3, available at https://doi.org/10.1016/j.iotech.2026.101600.

**Table 1:** Characteristics for all patients and according to disease stage

Variables	All patients	Stage I-III	Stage IV
**Number of cases, *n* (%)**	135 (100)	120 (89.9)	15 (11.1)
**Age at diagnosis (y), median (IQR)**	63 (54-71)	64 (54-72)	59 (53-71)
**Sex, *n* (%)**			
Men	81 (60)	70 (58.3)	11 (73.3)
Women	54 (40)	50 (41.7)	4 (26.7)
**Weight (kg), mean ± SD**	82.8 ± 16.5	82.2 ± 16.2	87.5 ± 18.4
**Duration of observation period (mo), median (IQR)**	45 (21-60)	51 (25-60)	18 (9-36)
**BRAF status, *n* (%)**			
Mutant	34 (25.2)	31 (25.8)	3 (20.0)
Wild type	19 (14.1)	17 (14.2)	2 (13.3)
Unknown	82 (60.7)	72 (60.0)	10 (66.7)
**Disease stage at diagnosis**			
I	7 (5.2)	7 (5.8)	
II	45 (33.3)	45 (37.5)	
III	68 (50.4)	68 (56.7)	
IV	15 (11.1)		

BRAF, human gene which encodes B-Raf protein; IQR, interquartile range; SD, standard deviation.

### Associations of patient characteristics and clinical parameters with BAT activity

**Figure 2:**
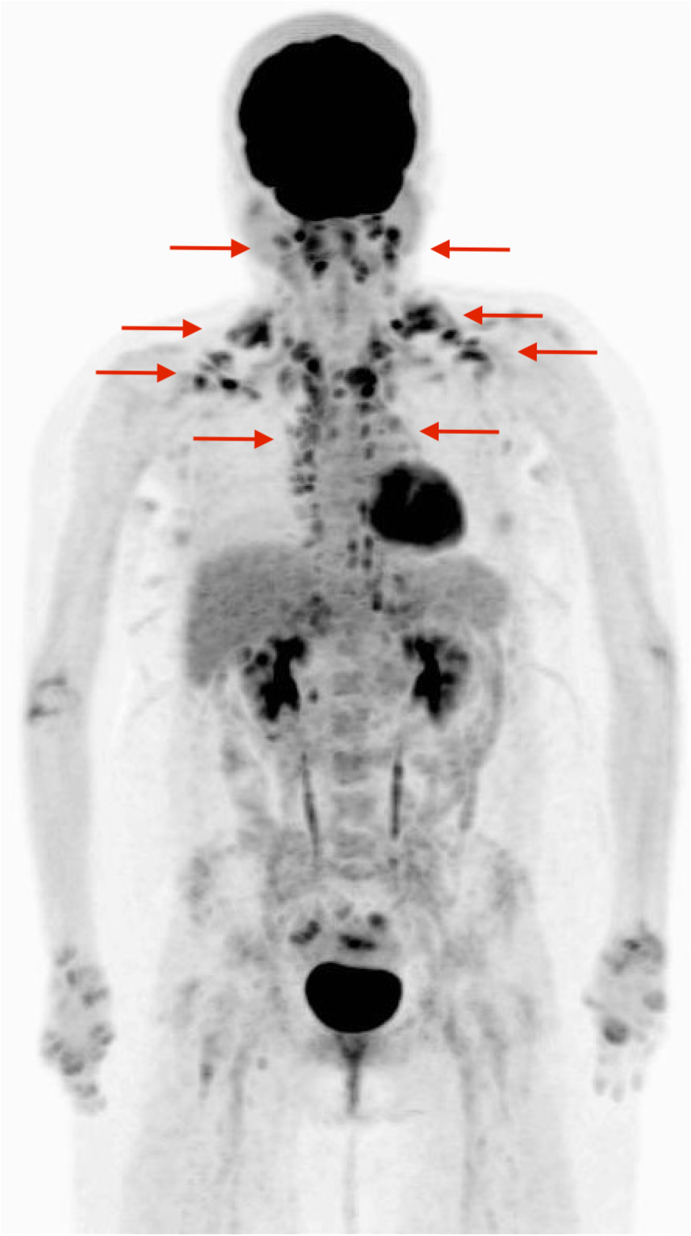
**Representative 2-fluoro-2-deoxy-D-glucose positron emission tomography-computed tomography (FDG–PET–CT) image of active brown adipose tissue (BAT).** Maximum-intensity projection positron emission tomography-computed tomography (PET–CT) image demonstrating increased [^18^F]2-fluoro-2-deoxy-D-glucose uptake in characteristic BAT deposits (red arrows) in the cervical, supraclavicular, and paravertebral regions.

BAT activity was measured independently by two investigators. Visualization of BAT activity in PET images in shown in Figure 2. Characteristics and the number of patients by BAT activity status at each threshold are shown in Table 2. Disease stage at diagnosis according to BAT activity status is shown in Table 3. High BAT activity (BAT_high_) was observed in 40 (29.6%), 24 (17.8%), and 19 (14.1%) patients at the 0.8, 1.0, and 1.2 g/ml thresholds, respectively. The median SUV_max_ was 0.78 g/ml (IQR 0.67-0.86, range 0.43-1.16). Women were more likely than men to have active BAT at the 0.8 threshold (*P* = 0.002) and at the 1.0 threshold (*P* = 0.043), whereas at the 1.2 threshold, the difference between the sexes was not significant (*P* = 0.086). In addition, women had a higher measured SUV_max_ than men (median 0.7941 versus 0.7330 g/ml; *z* = 3.562, *P* < 0.001). The differences in BAT volume between the sexes were not significant at any threshold (Mann–Whitney *U* test, *P* > 0.05 for all thresholds*;* Supplementary Table S4, available at https://doi.org/10.1016/j.iotech.2026.101600).

**Table 2:** Patient characteristics according to BAT status

Variables	All patients	BAT-negative	BAT_0.8_	BAT_1.0_	BAT_1.2_
Number of cases, *n* (%)	135 (100)	95 (70.4)	40 (29.6)	24 (17.8)	19 (14.1)
Age at diagnosis (y), median (IQR)	63 (54-71)	65 (54-72)	58 (49-71)	56 (49-72)	55 (44-65)
Sex (men : women), *n* (%)	81 (60)/54 (40)	65 (68)/30 (32)	16 (40)/24 (60)	10 (42)/14 (58)	8 (42)/11 (58)
Duration of observation period (mo), median (IQR)	45 (21-60)	49 (24-60)	39 (18-58)	39 (18-60)	39 (22-63)
BAT volume (mm^3^), median (IQR)			425 (87-4753)	855 (95-22 099)	4086 (98-18 603)

BAT, brown adipose tissue; IQR, interquartile range.

**Table 3:** Distribution of disease stage for all patients and by BAT positivity thresholds

Variables	I (*n* = 7), *n* (%)	II (*n* = 45), *n* (%)	III (*n* = 68), *n* (%)	IV (*n* = 15), *n* (%)	*P* value^a^
BAT_0.8_	5 (71.4)	12 (26.7)	20 (29.4)	3 (20.0)	0.101
BAT_1.0_	5 (71.4)	5 (11.1)	11 (16.2)	3 (20.0)	0.006
BAT_1.2_	3 (42.9)	4 (8.9)	10 (14.7)	2 (13.3)	0.133

BAT, brown adipose tissue.

a Comparisons of stage distribution between BAT-positive and BAT-negative at each threshold; Fisher–Freeman–Halton exact test.

BAT_high_ patients weighed less than those with low BAT activity (BAT_low_) at the 0.8 and 1.2 thresholds, while at the 1.0 threshold, the difference was not significant (Table 4). Additionally, a weak negative correlation between SUV_max_ and patient weight was observed [*r(133)* = −0.18, *P* = 0.038]. In BAT_high_ patients, no significant correlation between BAT volume and patient weight was observed (Spearman’s rank correlation, *P* > 0.05 for all thresholds; Supplementary Table S5, available at https://doi.org/10.1016/j.iotech.2026.101600).

**Table 4:** Patient weight according to BAT status

Threshold	BAT-positive, *n* (%)	Weight (kg), mean ± SD	BAT-negative, *n* (%)	Weight (kg), mean ± SD	Mean difference (95% CI)	*t*-statistic (*df*)	*P* value^a^
0.8	40 (30.0)	77.38 ± 15.7	95 (70.0)	85.04 ± 16.4	7.67 (1.63-13.70)	2.5 (133)	0.013
1.0	24 (17.8)	77.71 ± 16.2	111 (82.2)	83.86 ± 16.4	6.16 (−1.15 to 13.46)	1.7 (133)	0.098
1.2	19 (14.1)	75.26 ± 16.0	116 (85.9)	84.0 ± 16.3	8.74 (0.77-16.70)	2.2 (133)	0.032

BAT, brown adipose tissue; CI, confidence interval; SD, standard deviation.

a Independent samples *t*-test.

In BAT_high_ patients, no significant difference in BAT volume was observed between patients with metastatic and those with local disease at the time of PET–CT imaging at any threshold (Mann–Whitney *U* test, *P* > 0.05 for all thresholds*;* Supplementary Table S6, available at https://doi.org/10.1016/j.iotech.2026.101600).

Among the 135 patients, 34 presented with active disease and 82 with nonactive disease on PET–CT. A statistically significant association between active disease status on PET–CT and BAT_high_ was observed at the 0.8 and 1.0 thresholds, whereas at the 1.2 threshold, no significant association was observed (Table 5). No significant difference was observed in BAT volume between patients with active and those with nonactive disease at any threshold (Mann–Whitney *U* test, *P* > 0.05 for all thresholds; Supplementary Table S7, available at https://doi.org/10.1016/j.iotech.2026.101600). No significant difference was observed in WAT activity between patients with active and those with nonactive disease (mean 0.20 versus 0.20 g/ml, *t* = −0.48, *P* > 0.05).

**Table 5:** Crosstabulations of BAT status and disease activity on PET–CT

Threshold	BAT-positive, *n* (%)	BAT-negative, *n* (%)	Test statistic (c^2^ test or exact) (*df*)	*P* value
**0.8**				
Active disease	16 (44.4)	18 (22.5)	5.8^a^(1)	0.026^a^
No active disease	20 (55.6)	62 (77.5)		
Total, *N*	36	80		
**1.0**				
Active disease	11 (52.4)	23 (24.2)	6.6^a^(1)	0.016^a^
No active disease	10 (47.6)	72 (75.8)		
Total, *N*	21	95		
**1.2**				
Active disease	9 (56.2)	27 (27.0)	Exact	0.235^b^
No active disease	7 (43.8)	73 (73.0)		
Total, *N*	16	100		

BAT, brown adipose tissue; PET–CT, positron emission tomography-computed tomography.

a Chi-square test.

b Fisher’s exact test.

BRAF mutation status was available for 53 patients, of whom 34 had BRAF-mutant melanoma and 19 had BRAF-negative melanoma. During the observation period, metastatic disease occurred at any point in 68% of patients who underwent BRAF testing, compared with 45% of those who were not tested. Of the 53 patients with a BRAF mutation status, 18, 10, and 7 were classified as BAT_high_ at the 0.8, 1.0, and 1.2 g/ml thresholds, respectively. BRAF-mutant melanoma patients were more likely to have BAT_high_ at the 1.0 threshold (Fisher’s exact test, *P* = 0.009) and at the 1.2 threshold (Fisher’s exact test, *P* = 0.041) compared with BRAF wild-type patients, while at the 0.8 threshold, no significant difference was observed (chi-square test, *P* > 0.05; Supplementary Table S8, available at https://doi.org/10.1016/j.iotech.2026.101600).

### Prognostic significance of BAT activity

One hundred two patients had local disease at the time of PET–CT imaging. Of these, 61 were men, and 41 were women, with 28, 14, and 12 patients having BAT_high_ at the 0.8, 1.0, and 1.2 g/ml thresholds, respectively. The mean duration of the observation period was 47 months (SD 23, range 9-112). In this group, the median age at diagnosis was 63 years (IQR 53-70, range 19-83). During the observation period, 40 of these patients developed metastatic disease, with a median time from diagnosis to metastasis of 19 months (IQR 11-37, range 5-97). In this group, there was no significant association between BAT volume and disease stage at diagnosis at any threshold (Kruskal–Wallis test, exact *P* > 0.05 for all thresholds; Supplementary Table S9, available at https://doi.org/10.1016/j.iotech.2026.101600). BAT_high_ patients who did not develop metastatic disease during the observation period had larger BAT volumes at the 1.0 threshold (median 34 076.6 versus 391.2 mm^3^ in the metastatic group; *z* = 2.47, exact *P* = 0.012) and at the 1.2 threshold (median 20 589.2 versus 208.6 mm^3^ in the metastatic group; *z* = 2.19, exact *P* = 0.030) compared with BAT_high_ patients who developed metastatic disease. In contrast, at the 0.8 threshold (median 396.6 versus 369.5 mm^3^ in the metastatic group), there was no significant difference between the groups (Mann–Whitney *U* test, *P* > 0.05). Kaplan–Meier analysis revealed no statistically significant difference in relapse-free survival between BAT_low_ and BAT_high_ patients at any threshold (log-rank test, *P* > 0.05 for all thresholds). In the local disease population, a Cox proportional hazards regression analysis, adjusted for age, sex, and disease stage, was conducted to assess the effect of BAT_high_ on relapse-free survival. No statistically significant association between BAT_high_ and relapse-free survival was observed at any threshold (*P* > 0.05 for all thresholds; Supplementary Table S10, available at https://doi.org/10.1016/j.iotech.2026.101600).

Metastatic disease occurring before the analysed PET–CT scans was found in 33 patients, of whom 20 were men and 13 were women. Of these patients, 12, 10, and 7 were BAT_high_ at the 0.8, 1.0, and 1.2 g/ml thresholds, respectively. In this group, 13 deaths from any cause occurred during the observation period. The median duration of the observation period was 29 months (IQR 12-50, range 4-112), and the median survival time was 16 months (IQR 10-32, range 4-102). In this group, the mean age at diagnosis was 62 years (SD 13, range 29-82). Compared with BAT_high_ patients who died, BAT_high_ patients who survived the observation period had a higher measured BAT volume at the 0.8 threshold (median 3754.9 versus 86.9 mm^3^ in those who died, *z* = 2.28, exact *P* = 0.018) and at the 1.0 threshold (median 7620.1 versus 62.6 mm^3^ in those who died, *z* = 2.13, exact *P* = 0.038). At the 1.2 threshold, only one patient died during the observation period; thus, no statistically significant difference in BAT volume was observed between the groups (Mann–Whitney *U* test, *P* > 0.05). Kaplan–Meier analysis revealed no significant difference in melanoma survival between BAT_low_ and BAT_high_ patients at any threshold (Log-rank test, *P* > 0.05 for all thresholds). In the metastatic disease population, a multivariable Cox proportional hazards regression analysis, adjusted for age and sex, was conducted to assess the effect of BAT_high_ on patient survival. No significant association between BAT_high_ and patient survival was observed at any threshold (*P* > 0.05 for all thresholds; Supplementary Table S11, available at https://doi.org/10.1016/j.iotech.2026.101600).

## Discussion

Little is known about the role of BAT in melanoma progression and outcomes. Therefore, we conducted a retrospective registry study analysing PET–CT imaging from 135 melanoma patients to investigate whether disease characteristics, disease progression, and patient weight differ between BAT-positive and BAT-negative patients.

Our study showed that active melanomas are more likely to have active BAT than melanomas in patients without active disease. This association was statistically significant at the two lowest SUV thresholds in our study, whereas the highest SUV threshold (1.2 g/ml) did not reach statistical significance, possibly due to the small sample size. This is in line with previous research showing that BAT activity is greater in patients with active cancer compared with age-, sex-, and BMI-matched BAT-positive patients without active cancer.^15^ It has also been shown that BAT-positive patients with active cancer have higher BAT metabolic activity than those without active cancer.^14^ To explore whether this association could be explained by systemic factors such as metabolic stress or inflammation, we assessed WAT activity as an exploratory surrogate; however, no difference was observed between the groups. While this does not exclude confounding, it suggests that the association may not be solely attributable to generalised systemic effects.

A study by Bos et al.^15^ showed twofold greater BAT volumes in BAT-positive patients with active cancer than in those without active cancer in a study population of multiple types of solid tumour malignancies. In our study population, no statistically significant difference in BAT volume was observed between BAT_high_ patients with active and inactive disease. The discrepancy in results regarding BAT volume and disease status between our study and previous studies remains unclear.

In our study, active BAT was found in 29.6%, 17.8%, and 14.1% of patients at the 0.8, 1.0, and 1.2 g/ml thresholds, respectively. To our knowledge, no prior studies have investigated the prevalence of active BAT in melanoma patients. Cao et al.^26^ found that among patients with breast cancer, active BAT was present in 16.7% of patients overall, and in 25.6% of those under 55 years of age. In their control group, consisting of patients with mainly colorectal and lung cancer, the prevalence of active BAT was 5.2%.^26^ In a recent meta-analysis, Worku et al.^30^ concluded, based on pooled results from eight hospital-based studies, that the prevalence of active BAT among adults is 6.79%. Our results suggest that, in terms of the prevalence of active BAT, melanoma is comparable to breast cancer. It should be noted, however, that our lowest threshold of 0.8 g/ml most likely overestimates BAT_high_.

Current evidence suggests that noncancerous cells, including adipocytes, can alter cancer cell metabolism and affect processes involved in invasion, metastasis, and immune clearance.^31^ Mechanisms by which BAT could promote tumour growth and cancer progression have been suggested and studied; the most prominent is increased angiogenesis due to BAT activation. Increased angiogenic activity could, in turn, promote tumour dissemination and progression. It has been shown that implantation of tumour cells into BAT in mice results in accelerated tumour growth and increased angiogenesis,^32^ and that cold exposure-induced browning of WAT increases vascular density via angiogenesis.^18^ The study by Chu et al.^16^ of 132 patients with BAT-positive cancer found that BAT volume is a positive predictor of tumour recurrence and tumour-associated mortality, independent of other risk factors. In our study population, no meaningful relationship between BAT_high_ and disease stage at diagnosis was observed. Even though a statistically significant difference in disease stage distribution between BAT_high_ and BAT_low_ patients was observed at the 1.0 threshold, we deem this finding a false positive because the difference was only observed at this threshold. Furthermore, in our study, no association between BAT_high_ and survival or relapse-free survival was observed. Due to the small number of BAT_high_ patients, especially in subgroup analyses, no reliable evaluation of the effect of BAT volume on disease progression and melanoma-specific mortality could be conducted. However, among the BAT_high_ population, both patients with local disease who did not develop metastatic disease during the observation period and those with metastatic disease who survived had higher BAT volumes. This finding appears to be inconsistent with previous evidence. Another consideration is the accuracy and reliability of using FDG–PET–CT for assessing BAT volume, as this technique can only measure BAT that is metabolically active during the scan. Furthermore, several key factors, such as the partial volume effect and insulin resistance, introduce nontrivial uncertainty in quantifying metabolically active BAT with FDG–PET–CT. In summary, no prognostic effect of BAT status on melanoma outcomes was observed in our study. We could not analyse the effect of BAT volume on survival or relapse-free survival due to the larger number of BAT_high_ patients required for such an analysis, but a trend towards favourable outcomes and higher BAT volumes was noted.

Existing evidence in tumour-free subjects has shown that BAT positivity is associated with lower obesity parameters and BMI.^11^ In cancer, previous studies in mice have demonstrated an association between thermogenic activity in adipose tissue and CAC. Mouse models have shown that browning of WAT occurs in CAC at an early stage, before the onset of significant muscle and adipose wasting,^22^ and that suppressing thermogenic adipose tissue ameliorates adipose wasting in tumour-bearing mice.^22^^,^^23^ In their cohort of patients with cancer, Bos et al.^15^ observed that patients without active cancer had a positive association between abdominal fat deposits and BAT activity, whereas this association was not observed in patients with active cancer, suggesting that BAT plays a different role in modulating body composition depending on cancer activity. However, a recent study by Becker et al.^24^ casts doubt on the hypothesis that CAC is mediated by increased BAT activity. While their retrospective analysis of 13 461 PET scans confirmed an association between low BMI and BAT_high_, no association between cancer burden and BAT activity or BMI was found in their intra-individual longitudinal analysis of multiple PET scans from 238 patients.^24^ In our study, BAT-positive patients weighed markedly less than BAT-negative patients. We attribute this finding to the known association between BAT and low obesity parameters, which has been well demonstrated in the healthy adult population.^33^ No conclusion regarding the role of BAT in CAC can be drawn from these findings.

The BRAF mutation is a common oncogenic driver in melanoma, present in up to 50% of patients. The practices of BRAF mutation testing have evolved over the course of our study period. Before the introduction of BRAF pathway-targeted adjuvant therapy in 2020, testing was usually limited to metastatic cases; currently, BRAF testing is routinely conducted from stage IIC onwards.^34^ In our study, a statistically significant correlation was observed between BAT positivity at the 1.0 and 1.2 thresholds and the presence of confirmed BRAF mutations. Since BRAF testing is predominantly carried out in patients with advanced disease, those tested for BRAF mutations may have more active disease than those not tested. This is supported by the higher proportion of metastatic cases during the observation period among patients who underwent BRAF testing. However, because the disease stage at the time of BRAF testing was not recorded, the relationship between testing status and disease severity could not be fully characterised. We attribute the association between BRAF-mutant melanoma and BAT positivity to more advanced disease in patients with confirmed BRAF mutations. Currently, no evidence suggests a direct link between BRAF mutation positivity and BAT activity.

Our study had several limitations that need to be acknowledged. In addition to the retrospective nature of this study, a key limitation was the inability to apply LBM-corrected threshold values to identify BAT, which necessitated a three-stage universal threshold approach. Although the use of three predefined thresholds allowed us to assess the consistency of findings across different SUV cut-offs, it may limit direct comparability with studies using a single threshold or standardised LBM-adjusted criteria. Separate analyses were carried out for each threshold without adjustment for multiple comparisons, thereby increasing the risk of type I errors and incidental findings. Therefore, statistically significant threshold-dependent results should be interpreted with caution and considered exploratory. Furthermore, the number of BAT_high_ patients at the highest threshold (SUV_max_ > 1.2 g/ml) was relatively small, resulting in limited statistical power. As a consequence of the small number of BAT_high_ patients, no analyses of the effect of BAT volume on melanoma prognosis could be conducted. In addition, due to confounding factors and uncertainty in measuring perirenal and paravertebral adipose deposits, we decided to exclude these measurements from the analyses. This could, in turn, result in underreporting of BAT_high_. However, the correlation between SUV_max_ and key findings in this study, such as patient weight and sex differences, provides some validation of the methodology. Future studies on this topic would benefit from a multicentre approach, as combining data would increase statistical power.

As previously stated, several key factors introduce uncertainty in measuring BAT volume with FDG–PET–CT. Most importantly, with FDG–PET–CT, only BAT that is metabolically active during the scan can be measured. Furthermore, BAT deposits are highly heterogeneous in cellular composition, with large variation in the white adipocyte fraction. Combined with the partial volume effect, which is the dilution of the PET signal by surrounding tissues from foci near the resolution limit of the PET scanner, this results in an underestimation of BAT volume.^28^ Also, given that BAT activity is most commonly found in thin fat deposits located in the cervical and supraclavicular regions, the partial volume effect introduces nontrivial uncertainty in accurately measuring BAT volume at these locations. In addition, diabetes and insulin resistance affect FDG uptake in BAT. Studies have shown that in individuals with insulin resistance, glucose uptake in BAT is reduced, even though fatty acid uptake and oxygen consumption remain intact.^35^ Thus, FDG–PET–CT may underestimate BAT activity and volume in these patients.

Due to the retrospective use of FDG–PET–CT images taken as part of clinical practice rather than prospectively for research-specific PET–CT, no room-temperature measurements at the time of the PET–CT studies were obtained to control for cold-stimulus activation of BAT. Furthermore, the ambient temperature outside was left unadjusted. Patient tobacco and alcohol use, blood glucose level preceding PET–CT, and patient weight change in the months preceding PET–CT were not recorded or adjusted for in this study. The aforementioned factors that either promote or diminish BAT activity, and thus, if left unadjusted, weaken the strength and accuracy of the results.

Another limitation of this study was the use of different PET–CT imaging equipment over the observation period. However, this is unlikely to have introduced systematic bias, as imaging equipment in the clinical setting is assigned based on availability at any given time, effectively resulting in random allocation. Moreover, the uniform distribution of both BAT_high_ and BAT_low_ cases over the observation period, as visually confirmed by a dot plot, suggests that changes in the equipment did not affect the detection of BAT in this study population.

Another consideration is that the only measure investigated related to CAC was patient weight at the time of PET–CT imaging. A single measurement of patient weight is not sufficient to assess the presence of CAC; thus, no conclusion can be drawn from the results of this study between BAT activation and CAC in melanoma patients, although the findings point out a clear need for further research in this field, containing contradictory findings.

The strength of our study was the systematic and thorough analysis of each PET–CT image to assess BAT activity in a large dataset, a process that is time- and resource-intensive. The cohort consisted solely of patients with cutaneous melanoma and included a balanced number of women and men. Moreover, our clinical data were collected manually, thereby ruling out potential errors associated with the automated extraction of structural data from patient records.

In conclusion, patients with active melanoma at the time of PET–CT imaging were more likely to exhibit active BAT than those with no evidence of disease. No prognostic effect of BAT activity or volume on melanoma progression or survival was demonstrated in this cohort; accordingly, these findings have no immediate clinical applicability. Although higher BAT volumes were observed in subgroups of patients with more favourable outcomes, these findings were based on small subgroup analyses and should be considered exploratory. Further research on the subject is needed with a larger cohort of BAT_high_ patients in a prospective setting.
