# Supplementary material for: Association between brown adipose tissue activity and clinical outcomes in melanoma: a retrospective PET/CT analysis
Source: Immunooncol Technol. 2026 Jul 23;31:101600. doi: 10.1016/j.iotech.2026.101600 (PMC13427674; doi:10.1016/j.iotech.2026.101600)
Supplement: Supplementary Tables S1-S11 [file mmc1.docx]

Supplement 1. Adjuvant therapies received by patients with local disease at time of PET/CT.

| **Adjuvant treatment** | **N (=102)** |
| --- | --- |
| Interferon | 8 |
| Interferon + Radiotherapy | 2 |
| Radiotherapy | 1 |

Supplement 2. Treatments received by patients with metastatic disease at time of PET/CT.

| **Treatment** | **Patients (% of treatments overall)** |
| --- | --- |
| Pembrolizumab | 10 (20.8) |
| Dabrafenib + Trametinib | 7 (14.6) |
| Interferon | 3 (6.3) |
| Nivolumab | 3 (6.3) |
| Cisplatin + Dacarbazine + Interferon | 2 (4.2) |
| Cisplatin + Etoposide | 2 (4.2) |
| Ipilimumab | 2 (4.2) |
| Lomustine + Bleomycin + Vincristine + Dacarbazine + Interferon | 2 (4.2) |
| Temozolomide + Vincristine + Lomustine | 2 (4.2) |
| Vemurafenib | 2 (4.2) |
| Paclitaxel + Carboplatin | 2 (4.2) |
| Cisplatin + Fluorouracil | 1 (2.1) |
| Dacarbazine | 1 (2.1) |
| Dacarbazine + Interferon | 1 (2.1) |
| Encorafenib + Binimetinib | 1 (2.1) |
| Imatinib | 1 (2.1) |
| Ipilimumab + Nivolumab | 1 (2.1) |
| Isolated limb perfusion | 1 (2.1) |
| Lomustine + Bleomycin + Vincristine + Dacarbazine | 1 (2.1) |
| Nivolumab | 1 (2.1) |
| NKTR-214 + Nivolumab | 1 (2.1) |
| Temozolomide + Vincristine + Lomustine + Interferon | 1 (2.1) |

Supplement 3. Number of patients by number of treatment courses for patients with metastatic disease at time of PET/CT.

|  | **One line of treatment** | **Two lines of treatment** | **Three lines of treatment** | **Four lines of treatment** |
| --- | --- | --- | --- | --- |
| **Number of patients** | 27 | 11 | 7 | 3 |

Supplement 4. BAT volume across sex.

| **Threshold level** | **Median (IQR) BAT volume in mm3** |  | **Z** | **P-value^a^** |
| --- | --- | --- | --- | --- |
|  | Male | Female |  |  |
| **Threshold 0.8** | 348 (69-8839) | 474 (87-4753) | -0.400 | 0.692 |
| **Threshold 1.0** | 432 (58-18494) | 1492 (168-25577) | -0.293 | 0.796 |
| **Threshold 1.2** | 2743 (90-15343) | 4086 (174-20589) | -0.413 | 0.717 |

a: Mann-Whitney U-test

| **Threshold level** | **Spearman correlation coefficient** | **P-value** |
| --- | --- | --- |
|  |  |  |
| **Threshold 0.8** | -0.113 | 0.488 |
| **Threshold 1.0** | -0.259 | 0.222 |
| **Threshold 1.2** | -0.096 | 0.697 |

Supplement 5. Correlation between BAT-volume and patient weight

| **Threshold level** | **Median (IQR) BAT volume in mm3** |  | **Z** | **P-value^a^** |
| --- | --- | --- | --- | --- |
|  | Metastatic | Local disease |  |  |
| **Threshold 0.8** | 474 (102-21802) | 369 (84-4143) | -0.709 | 0.493 |
| **Threshold 1.0** | 619 (57-16778) | 2,703 (179-27293) | -0.878 | 0.403 |
| **Threshold 1.2** | 814 (98-16039) | 4,651 (117-20093) | -0.254 | 0.837 |

Supplement 6. BAT-volume between metastatic and local disease at time of PET/CT

a: Mann-Whitney U-test, exact.

Supplement 7. BAT-volume between active and non-active disease at time of PET/CT

| **Threshold level** | **Median (IQR) BAT volume in mm3** |  | **Z** | **P-value^a^** |
| --- | --- | --- | --- | --- |
|  | Active | Non-active |  |  |
| **Threshold 0.8** | 348 (84-3577) | 440 (87-12975) | -0.764 | 0.459 |
| **Threshold 1.0** | 196 (44-13300) | 8,014 (440-61416) | -1.760 | 0.085 |
| **Threshold 1.2** | 814 (98-16039) | 5,278 (2130-54711) | -1.323 | 0.210 |

a: Mann-Whitney U-test, exact.

| **Threshold level** |  | **BAT-positive *n*** | **BAT-negative *n*** | **Test statistic**  **(*χ*2 or exact) (df)** | ***P*-value** |
| --- | --- | --- | --- | --- | --- |
| **Threshold 0.8** | Mutated BRAF *n* | 14(41.2%) | 20(58.8%) | 2.201 (1) | 0.138^a^ |
|  | Wildtype BRAF *n* | 4(21.1%) | 15(78.9%) |  |  |
|  | Total *n* | 18 | 35 |  |  |
| **Threshold 1.0** | Mutated BRAF *n* | 10(29.4%%) | 24(70.6%) | exact | 0.009^b^ |
|  | Wildtype BRAF *n* | 0(0.0%) | 19(100.0%) |  |  |
|  | Total *n* | 10 | 43 |  |  |
| **Threshold 1.2** | Mutated BRAF *n* | 7(20.6%) | 27(79.4%) | exact | 0.041^b^ |
|  | Wildtype BRAF *n* | 0(0.0%) | 19(100.0%) |  |  |
|  | Total *n* | 7 | 46 |  |  |

Supplement 8. Crosstabulations of BAT status and BRAF mutation status.

a: Chi-Square test. b: Fisher’s exact test

Supplement 9. Comparisons of BAT-volume between disease stage in the local disease at time of PET/CT population.

| **Threshold level** | **Disease stage** | **N** | **Median (min-max) BAT-volume in mm3** | **H** | **P-value^a^** |
| --- | --- | --- | --- | --- | --- |
| **Threshold 0.8** | I | 3 | 348 (209-1,314) |  |  |
|  | II | 7 | 304 (21-64,633) | 0.342 | 0.855 |
|  | III | 18 | 391 (21-236,885) |  |  |
| **Threshold 1.0** | I | 3 | 43 (42-668) |  |  |
|  | II | 2 | 32,077 (7,954-56,200) | 4.568 | 0.095 |
|  | III | 9 | 4,738 (130-222,498) |  |  |
| **Threshold 1.2** | I | 1 | 209 (209-209) |  |  |
|  | II | 2 | 26,514 (5,216-47,812) | 1.239 | 0.642 |
|  | III | 9 | 4,086 (43-206,242) |  |  |

a: Kruskall-Wallis test, exact

| **Threshold level** | **Variable** |  | **Adjusted HR (95% CI)** | **P-value** |
| --- | --- | --- | --- | --- |
| **Threshold 0.8** | Sex | Male | Reference |  |
|  |  | Female | 0.87 (0.44-1.73) | .699 |
|  | Age at diagnosis |  | 1.02 (1.00-1.05) | .106 |
|  | Disease stage group | I-II | Reference |  |
|  |  | III | 1.21 (0.62-2.36) | .585 |
|  | BAT | Negative | Reference |  |
|  |  | Positive | 1.90 (0.99-3.66) | .054 |
| **Threshold 1.0** | Sex | Male | Reference |  |
|  |  | Female | 0.97 (0.49-1.91) | .930 |
|  | Age at diagnosis |  | 1.02 (1.00-1.05) | .102 |
|  | Disease stage group | I-II | Reference |  |
|  |  | III | 1.23 (0.62-2.42) | .556 |
|  | BAT | Negative | Reference |  |
|  |  | Positive | 1.51 (0.68-3.34) | .308 |
| **Threshold 1.2** | Sex | Male | Reference |  |
|  |  | Female | 0.95 (0.48-1.87) | .883 |
|  | Age at diagnosis |  | 1.02 (1.00-1.05) | .107 |
|  | Disease stage group | I-II | Reference |  |
|  |  | III | 1.18 (0.61-2.31) | .625 |
|  | BAT | Negative | Reference |  |
|  |  | Positive | 1.30 (0.54-3.15) | .559 |

Supplement 10. Multivariable Cox proportional hazards model for disease-free survival among patients with local disease at time of PET/CT.

Supplement 11. Cox proportional hazards model for melanoma survival among patients with metastatic disease at time of PET/CT.

| **Threshold level** | **Variable** |  | **Adjusted HR (95% CI)** | **P-value** |
| --- | --- | --- | --- | --- |
| **Threshold 0.8** | Sex | Male |  |  |
|  |  | Female | 0.59 (0.15-2.24) | .435 |
|  | Age at diagnosis |  | 1.02 (0.97-1.07) | .516 |
|  | BAT | Negative |  |  |
|  |  | Positive | 1.51 (0.42-5.44) | .525 |
| **Threshold 1.0** | Sex | Male |  |  |
|  |  | Female | 0.60 (0.14-2.53) | .486 |
|  | Age at diagnosis |  | 1.02 (0.97-1.07) | .511 |
|  | BAT | Negative |  |  |
|  |  | Positive | 1.36 (0.32-5.77) | .674 |
| **Threshold 1.2** | Sex | Male |  |  |
|  |  | Female | 0.99 (0.28-3.44) | .982 |
|  | Age at diagnosis |  | 1.02 (0.96-1.08) | .477 |
|  | BAT | Negative |  |  |
|  |  | Positive | 0.25 (0.03-2.13) | .207 |
